# Evolution of extravascular implantable cardioverter-defibrillator therapy for ventricular arrhythmias

**DOI:** 10.1016/j.hroo.2022.09.021

**Published:** 2022-11-13

**Authors:** Hans Römers, Vincent van Dijk, Lucas Boersma

**Affiliations:** ∗ Department of Cardiology, St Antonius Hospital, Nieuwegein, the Netherlands; † Department of Cardiology, Amsterdam University Medical Centers, University of Amsterdam, Amsterdam, the Netherlands

**Keywords:** S-ICD, Extravascular, Arrhythmias, Pacing, EV-ICD

## Abstract

Implantable cardioverter-defibrillators have become an established therapy for the prevention of sudden cardiac death due to life-threatening ventricular arrhythmias in the last decades. In all those years, the use of transvenous leads has proven to be the most vulnerable part of the system. The development of the completely subcutaneous implantable cardioverter-defibrillator opened a new era of device therapy outside of the vascular system. The next step, enabling extravascular devices with the option of antitachycardia pacing, is just around the corner. This may become an important option for all patients without a bradycardia pacing indication that are in need for antitachycardia pacing because of monomorphic ventricular tachycardia.

**Key Findings**

▪ The subcutaneous implantable cardioverter*-*defibrillator has become a reliable alternative to transvenous implantable cardioverter*-*defibrillator therapy.
▪ Two new developments are under investigation, opening new possibilities.
▪ A subcutaneous implantable cardioverter*-*defibrillator combined with the Empower LP provides the extravascular implantable cardioverter*-*defibrillator with pacing treatment options.
▪ The extravascular implantable cardioverter*-*defibrillator offers extravascular pacing in a new concept, and early studies are promising.

## Introduction

Since the development of the implantable cardioverter-defibrillator (ICD) by Mirowski and colleagues in the 1970s, ICD therapy evolved from an abdominal shock box with epicardial patches to a much smaller device with transvenous implantable leads offering full pacing and defibrillation options for primary and secondary prevention indications.^1^ Over time, numerous trials have been performed that have led to new guidelines with a class I indications for preventing sudden cardiac death.^2^, ^3^, ^4^, ^5^ In all those years, the transvenous leads have repeatedly shown that they are prone to develop mechanical defects due to motion and mechanical stress over time. Subclavian crush is the mechanical factor that is responsible for up to 40% of failures after 5 years’ follow-up (FU).^6^ Furthermore, the occurrence of primary infections of the pocket or secondary infections with lead endocarditis can lead to necessitating lead extractions with a high rate of complications or even death.^7^ There have also been several lead design issues such as with the Sprint Fidelis (Medtronic, Minneapolis, Minnesota) and Riata (St. Jude Medical, St. Paul, Minnesota) that have caused significant morbidity and distress in patients. For this reason, alternative options that offer defibrillation while the device is positioned outside the heart and vasculature have been developed. The completely subcutaneous ICD (S-ICD) with the device in a left lateral thoracic position and the shock lead parallel to the sternum was the first of its kind and has become a reliable alternative to transvenous ICD (TV-ICD) therapy. In the last decade, several publications have reviewed the technical aspects of the S-ICD,^8^^,^^9^ which appears to have solved the intravascular aspect but not all lead issues.^10^ Moreover, new devices are just around the corner that may offer options for (limited) bradycardia pacing as well as for antitachycardia pacing (ATP).

## Current data on S-ICD

After Bardy and colleagues^10^ published the landmark study in 2010 on the efficacy of the S-ICD, several trials were performed to evaluate the efficacy and safety of the S-ICD. In the investigational device exemption trial, 321 patients had a complication-free rate of 99% during a 6-month FU.^11^ All but 1 spontaneous ventricular arrhythmia (VA) events were successfully converted by the ICD, while 1 monomorphic ventricular tachycardia (VT) converted spontaneously. Inappropriate shock (IAS) rate was rather high, at 13.1%, after 1-year FU in this trial. The Evaluation oF Factors ImpacTing CLinical Outcome and Cost EffectiveneSS of the S-ICD (EFFORTLESS) study,^12^ included 985 patients with a more than 5-year FU. The IAS rate after 3-year FU was 11.7% and after 5-year FU was 16.9%. Cardiac T-wave oversensing (68.3%) was the main reason for IAS, whereas 18.7% were due to supraventricular tachycardia (SVT) or atrial fibrillation events and 16.8% were for noncardiac origin. The complication rate after 1 year was 8.4%. A total of 16% of the patients experienced appropriate shock therapy in 5 years, and the shock efficacy after 5 years of FU is still reliably high at 98%.

A Dutch cohort was assembled in the first years of S-ICD therapy, and a recent publication on 6-year FU data after implantation showed a low a complication rate of 3% without lead failure and a low annual IAS rate.^13^ Extraction of the S-ICD system was performed in 10 of 118 patients, of whom 8 had an ICD-related infection. All systems were extracted without complications.

Patient characteristics in these trials were somewhat different from most classic TV-ICD studies. The mean age was lower, the left ventricular ejection fraction was higher, and there were less comorbidities. New studies in more common populations with guideline indications for ICD therapy were therefore needed.

The first and so far only randomized clinical trial comparing the S-ICD with the TV-ICD is the PRAETORIAN trial^14^ including 876 patients with a class I or IIa indication for primary or secondary prevention, an average age of 63 years, median left ventricular ejection fraction of 30%, and FU duration of 49 months. Using a noninferiority design, with a composite primary endpoint of IAS and device complications, proved that the S-ICD for patients with an ICD indication without a pacing need was noninferior to TV-ICD (*P* = .01). In the S-ICD group, fewer lead-related complications were seen (1.4% vs 6.6%). IAS events occurred less in the TV-ICD population (29 [7.3%] vs 41 [9.6%]). The cause of IAS events in the S-ICD population was mainly cardiac oversensing (24 vs 2), while for the TV-ICDs the majority were due to SVT or atrial fibrillation (27 vs 11). A higher number of all causes of mechanical lead complications occurred in the TV-ICD group (32 vs 13). In the PRAETORIAN trial, the inability to pace was observed in 68 events of monomorphic VT event in S-ICD group.^14^ These events were seen above (57 events) and below (11 events) cutoff zones. VT events in the zones were treated by shock therapy, which could have been avoided if ATP therapy was available. The PAINFREE study showed that ATP is successful in up to 80% in terminating fast VT events.^15^ The VT below cutoff zone received a ICD shock due to oversensing. In 91% of those events, the SMART Pass filter was not active or unavailable at first occurrence off these events, which could alleviate such inappropriate therapy. Overall, the conclusion was that the S-ICD is a good alternative to the TV-ICD for all patients without a pacing indication.

## Drawbacks of the S-ICD

The higher rate of IAS events in comparison with the TV-ICD, and the inability to pace the heart, have long been barriers for S-ICD therapy adoption. The higher potential for inappropriate shock requires a thorough electrocardiography screening process before implantation to avoid cardiac oversensing, most commonly of the T-wave. In addition, new algorithms were incorporated in next-generation S-ICD devices. The first algorithm for reducing IAS was introduced in 2015,^16^ using the morphological characteristics of the QRS to reduce charge decision by ruling out T-wave oversensing. Approximately 39.8 ± 11.4% reduction of IAS was achieved with the new algorithm without a negative effect on detection of VA or SVT events.^16^ The subsequent SMART Pass filter to reduce T-wave oversensing was evaluated by Theuns and colleagues,^17^ showing 68% reduction of the IAS burden.

The recently published UNTOUCHED trial evaluated the occurrence of IAS rates in primary prevention patients with the second- and third-generation S-ICD devices, with high-rate cutoff points for therapy and SMART Pass algorithm capability. Annual IAS rate was very low (3.1% overall), while the third-generation EMBLEM devices had an even lower IAS rate of 2.4%.^18^ Patients with the SMART Pass algorithm enabled had the lowest IAS rate. These IAS rates are as good as or even better than the Multicenter Automatic Defibrillator Implantation Trial–Reduce Inappropriate Therapy (MADIT-RIT) trial outcomes that are currently considered to be the standard for modern ICD therapy.^18^^,^^19^

## Defibrillation threshold testing

During ICD implantation, it was common practice to test the defibrillation threshold (DFT). This may be associated with a higher risk of complications like inability to convert induced VF to normal rhythm, which resulted in prolonged resuscitation, stroke, or mortality. Several studies have shown that DFT testing does not convey a long-term benefit to predict shock effectiveness or mortality and is only useful in selected patients. DFT testing in S-ICD implantations has a class I indication due to the extrathoracic position of the lead, which is associated with a higher threshold and in need for a higher energy level. The S-ICD can deliver twice the amount of energy compared with the TV-ICD. The development of the PRAETORIAN score by Quast and colleagues^20^ provided an algorithm capable of predicting the efficacy of DFT test. It is based on the position of the can and lead combined with a lateral and anteroposterior chest radiograph. Retrospective validation of this score has resulted in a sensitivity and specificity of 95%. A low PRAETORIAN score predicts a high probability of a successful DFT test. In DFT tests, a success rate of 99.8% was achieved. The randomized PRAETORIAN DFT trial, designed to prospectively evaluate if DFT testing is still required when lead and can are placed adequately, has started enrollment, but outcomes are not expected before 2023.

## S-ICD and the Empower LP system

Two new developments are under investigation to enable non–TV-ICD with pacing options. The combination of an S-ICD with the Empower LP system from Boston Scientific (Marlborough, Massachusetts) and the extravascular ICD (EV-ICD) concept from Medtronic. Both are non–TV-ICD systems but with a different approach for the ATP delivery method.

**Figure 1:**
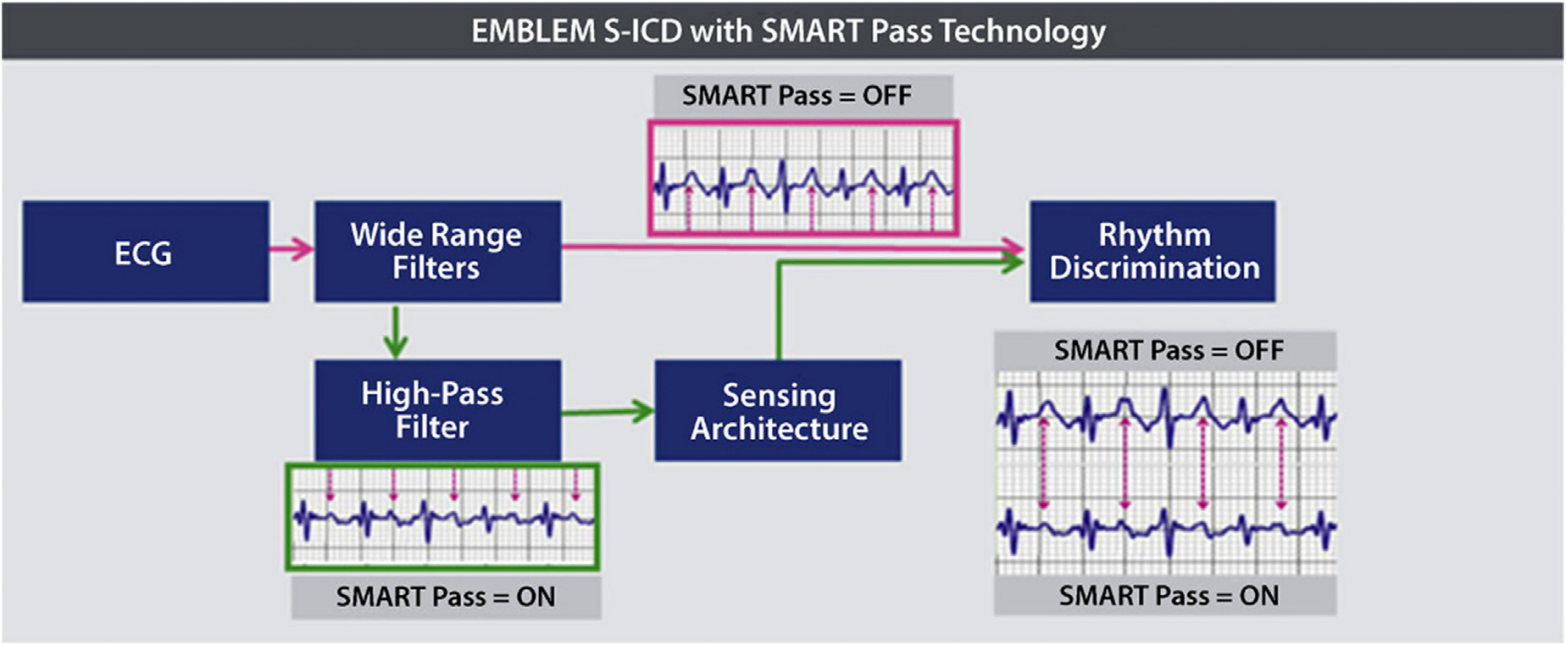
Introduction of the SMART Pass algorithm led to improved rhythm discrimination. Material provided courtesy of Boston Scientific. ECG = electrocardiography; S-ICD = subcutaneous implantable cardioverter-defibrillator.

Boston Scientific has been working on combining the S-ICD with a leadless cardiac pacemaker (LCP), the Empower, into a modular system (Figure 1). To make this work, the communication between both systems is essential.^21^ Animal studies were performed to prove that the perpendicular position of the LCP to the lead was the optimal position for effective unidirectional communication.^21^, ^22^ The creation of a communication vector is established by a burst of consecutive signals that are sent from the S-ICD coil toward the S-ICD can. The LCP must be placed in this created field to be able to detect this signal as a voltage difference between the 2 sensing electrodes (distal and proximal) placed at the end of the LCP. When the S-ICD detects a VA episode that meets the VT criteria for ATP, it will send these signals toward the LCP. After recognition, the LC will deliver ATP.^21^ The first study, published in 2016, showed that ATP successfully terminated the VT. In the following study involving 40 animals, the consistency of communication for sensing and delivering ATP was achieved.^23^ Shock therapy did not interfere with adequate sensing or pacing functions of the LCP. No trials have been performed in humans yet, but cases of combining S-ICD with a TV pacemaker have been published^24^ but without the possibility of communication and interaction between the 2 devices. This might not be suitable for all patients but for those who develop a pacing indication this could be a great alternative. To evaluate this new therapy option, the large MODULAR clinical trial has recently started enrollment.

## The substernal EV-ICD strategy

**Figure 2:**
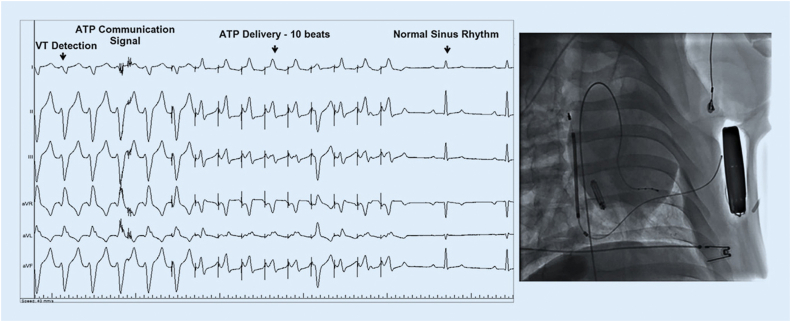
Subcutaneous implantable cardioverter-defibrillator and leadless cardiac pacemaker working together to provide antitachycardia concept for a simulated ventricular tachycardia (VT). ATP = antitachycardia pacing. Reprinted with permission from Tjong et al.^23^

The EV-ICD is a different approach to adding extravascular pacing options by implanting the lead substernal instead of the subcutaneous position of the S-ICD lead. Its conception and primary evaluation process was recently described by Thompson and colleagues.^25^ The pulse generator is in a similar lateral position as the S-ICD (Figure 2). The benefit of this position is that the lead is placed direct above the right ventricle, assuring a good sensing and pacing location. The first successful substernal defibrillation testing was described by Tung and colleagues.^26^ There are some case reports describing this method used in specific clinical circumstances.^27^ The development of the EV-ICD concept was done with 2 parallel main questions, the cardiac pacing feasibility and the sensing detection and defibrillation feasibility. The Substernal Pacing Acute Clinical Evaluation (SPACE) study focused on the pacing capabilities. In 18 (69%) of 26 patients, pacing across all vectors was possible with 5.8 ± 4.4 V with a pulse width of 10 ms.

Unipolar (2.98–4.11 mV) and bipolar (0.83–3.95 mV) R-wave amplitude detection was sufficient for development of algorithms for arrhythmia detection with a ventricular origin.^28^

Animal testing for the defibrillation capability showed a 1.5 times higher defibrillation threshold than normal TV-ICD thresholds (15 J vs 22 J).

The first-in-human Acute Extravascular Defibrillation, Pacing, and Electrogram (ASD) study included 14 patients for defibrillation testing. In 13 of 14 patients, the VF episode was terminated with a 35-J single shock after detection (18.4 ± 5.6 seconds) and a shock impedance of 98.1 ± 19.3 Ω.

**Figure 3:**
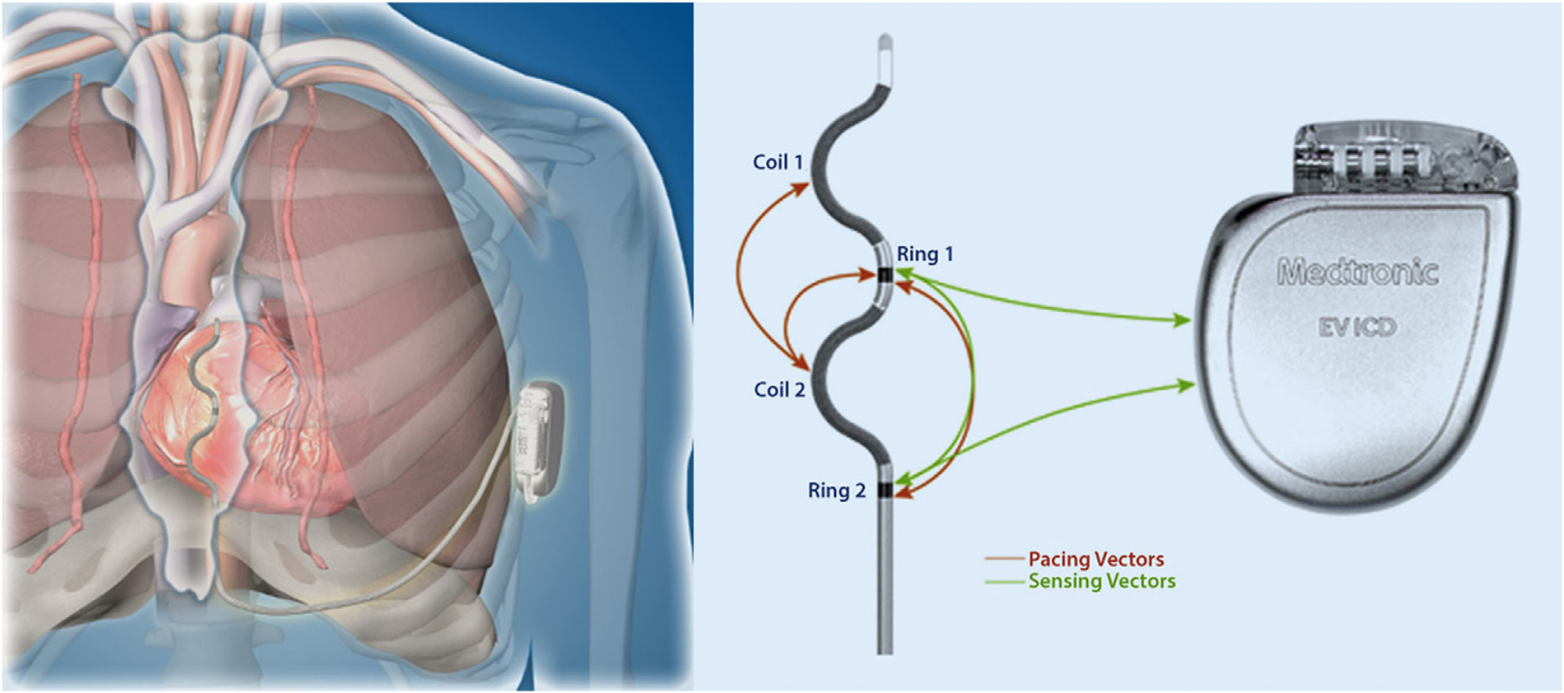
Schematic drawing of the position of the extravascular implantable cardioverter-defibrillator (EV-ICD) lead and picture of the EV-ICD device and epsilon-shaped EV-ICD lead with the 2 coils and 2 electrodes and the different pacing vectors.^31^ Reprinted with permission from Boersma et al.^30^

In the following acute ASD 2 study, a temporary substernal lead was implanted and a patch or active can emulator was used in the lateral position.^29^ The lead design with an epsilon-shaped body containing 2 shock coils to the right and 2 ring electrodes to the left (proximal and mid) for sensing proved to be feasible both pacing and defibrillation (Figure 3).^30^ A total of 128 ventricular events in 69 patients were induced (70 being VF), of which 104 (81%) were terminated with a 30-J shock. Pacing capabilities were also tested and were found successful at low-voltage (2-ms pulse width up to 8 V, 82% capture) or high-voltage (10-ms pulse width up to 40 V, 97% capture) output in up to 3 different vector settings. Proper lead placement was successful in 84% at first attempt and an average placement time of 12 minutes with a maximum of 4 attempts.

The first study with a chronical implanted EV-ICD based on the Medtronic Evera ICD platform was recently published.^31^ The Medtronic Evera ICD has a 40-J shock capability, which is only half of the maximum output of an S-ICD and also with a volume size that is half of the S-ICD, making it more comfortable for the patient and more easy to implant, with potentially better longevity. In 20 of 26 patients, a chronic implant was achieved. Mean sensing of the R-wave was 3.4 ± 2.0 mV and mean threshold was 5.4 ± 2.0 mV. A total of 95% of patients could be paced at <10 V. Sensing ventricular events at 0.3-mV sensitivity or higher was achieved in all patients. A mean shock energy of 15 J was achieved with a safety margin of 10 J or more in 90% of the patients.

Recently, the first results of the EV-ICD CE/investigational device exemption trial reported the outcomes of 299 patients that received a fully implanted device.^32^ After FU for at least 6 months for all patients, the 2 predefined primary endpoints were both met. The efficacy of the defibrillation testing for induced VAs was 98.7%, which well exceeded the 84% target that was set based on prior TV-ICD and S-ICD trial outcomes. The primary safety outcome showed freedom of major system or procedure-related complications of 92.6% at 6 months, which was also significantly better than the predefined target. Spontaneous arrhythmic events (n = 66) were treated with a shock therapy with a first shock efficacy of 78%, while ATP successfully terminated 32 (70%) of 46 episodes in 10 patients. The pause-prevention pacing therapy could not be turned on for all patients due to not tolerated pacing sensations. During FU, 7 episodes of asystole were noted and successfully treated with up to 19 pacing pulses delivered.

These positive results for efficacy and safety came along with a rather high rate of inappropriate therapy (9.7%) in 81 arrhythmia events with 118 shocks in 29 patients. The main causes were P-wave oversensing (34 events), lead noise (19 events), and atrial arrhythmia (11 events). New algorithms preventing inappropriate are available but have not been clinically studied. An infection rate of 1.3% was very low and similar to the S-ICD population, and all explants were performed without any major complication and no sign of mediastinitis or systemic infection.

Some comments may be made about this new lead design and implant location. Tunneling this lead in the substernal position requires additional training of implanting physicians, although the space directly under the sternum is well known to thoracic surgeons. It contains no major vessels or organs, but there is a risk of entering the pericardium, pleural space, or abdominal space. In the ADS 2 trial, 1 patient suffered from an asystolic event 48 hours after dismissal from the hospital and died of an undetermined origin. Another patient had a tamponade due to the accidental tunneling in the pericardium. Despite rapid pericardiocentesis, the patient did not survive due to the secondary complications 3 days later. After these events, training requirements have become even more stringent, and use of extensive imaging (Computed tomography) for extra guiding during tunneling was introduced. Ongoing clinical studies may shed more light on unfavorable effects of this novel implantation technique and lead design. The development of EV-ICD platforms does provide new treatment options for patients. From the first S-ICD to the new studies with the S-ICD and the EV-ICD, avoiding the vascular system is now becoming feasible for a wider population of patients in need of sudden cardiac death prevention. The new options for adding ATP to the extravascular system will broaden the indication area for these devices. Bearing in mind that these systems also have their limitations, the future will show the way that we should go.

## Conclusion

With the introduction of the S-ICD, the field of extracardiovascular defibrillation systems has opened to develop next-generation solutions to overcome some of the limitations of not being able to pace the heart with an intracardiac lead. Smart communication between the S-ICD and a novel LCP enables both ATP as well as bradycardia pacing options. The EV-ICD with a substernal lead may also fulfill the need for ATP, although currently bradycardia pacing options are limited. In the coming years, the outcomes of larger trials with these prototypes will show their effectiveness and safety, which will determine how widespread adoption may become to serve a wide population of patients in need of sudden cardiac death prevention.
